# The increase of philanthropic entities in the SUS: what does this scenario reveal?

**DOI:** 10.11606/s1518-8787.2023057004720

**Published:** 2023-06-02

**Authors:** João Felipe Marques da Silva, Brígida Gimenez Carvalho, Adelyne Maria Mendes Pereira, Elisabete de Fátima Almeida Nunes, Fernanda de Freitas Mendonça, Stela Maris Lopes Santini, Silvia Karla Azevedo Vieira Andrade, Edinalva de Moura Ferraz

**Affiliations:** I Universidade Estadual de Londrina Programa de Pós Graduação em Saúde Coletiva Londrina PR Brasil Universidade Estadual de Londrina. Programa de Pós Graduação em Saúde Coletiva. Londrina, PR, Brasil; II Universidade Estadual de Londrina Centro de Ciências da Saúde Departamento de Saúde Coletiva Londrina PR Brasil Universidade Estadual de Londrina. Centro de Ciências da Saúde. Departamento de Saúde Coletiva. Londrina, PR, Brasil; III Fundação Oswaldo Cruz Escola Nacional de Saúde Pública Sergio Arouca Departamento de Administração e Planejamento em Saúde Rio de Janeiro RJ Brasil Fundação Oswaldo Cruz. Escola Nacional de Saúde Pública Sergio Arouca. Departamento de Administração e Planejamento em Saúde. Rio de Janeiro, RJ, Brasil; IV Fundação Oswaldo Cruz Escola Nacional de Saúde Pública Sergio Arouca Programa Pós-Doutorado Junior - Inova Fiocruz Rio de Janeiro RJ Brasil Fundação Oswaldo Cruz. Escola Nacional de Saúde Pública Sergio Arouca. Programa Pós-Doutorado Junior - Inova Fiocruz. Rio de Janeiro, RJ, Brasil

**Keywords:** Voluntary Health Agencies, legislation & jurisprudence, Fund Raising, Health Management, Unified Health System, Public Policy

## Abstract

The article analyzes aspects of the change in the legal nature of private healthcare from “for-profit” to “non-profit” entities. It is an exploratory research, supported by the policy analysis framework, focusing on secondary data from the *Cadastro Nacional de Estabelecimentos de Saúde* (National Registry of Health Facilities – CNES) from 2012 to 2020 and a case study. The results show an increase in these entities in all regions of the country and evidence that they behave like profit-oriented entities. The change in legal nature hides a broader process of implicit commodification of healthcare services, encouraged by state policies and related to exemptions provided by law.

## INTRODUCTION

In Brazil, non-profit private institutions play a significant role in providing specialized healthcare^1^ , being part of the complex public-private relationship within the Brazilian Unified Health System (SUS). This relationship is characterized by power asymmetries in decision-making and governance of health policy^1 , 2^ and has been historically supported by the state’s expansion of the private sector. This support may take the form of commodification of healthcare services, using direct and indirect subsidies, such as tax exemptions and fiscal incentives applied to these entities^3^ .

Commodification is related to the increase in the private sector’s influence, through their direct participation as a healthcare entities, and the adoption of private system management, remuneration, and organization principles^4^ . These characteristics seem to encourage the change in legal nature of private healthcare entities from “for-profit” to “non-profit” status.

This process has led to new public-private relationships with neo-patrimonialistic characteristics, which benefit both the State and the market. Furthermore, counter-reform policies of the 1990s encouraged the strengthening of modalities that benefit from the legal prerogatives of non-profit entities^5^ .

This study focuses on the change in legal nature of healthcare entities over the past decade. The study is based on two main arguments: the first refers to a transition from private “for-profit” to “non-profit” entities, which may represent a trend in changing public-private relationships in healthcare in Brazil; and the second, related to the former, concerns the commodification mechanisms that may be employed through this transition. This study aims to analyze and identify characteristics of this phenomenon to stimulate and expand the debate, as well as propose and encourage further research on the topic.

## METHODS

This study is a subset of a doctoral thesis that aimed to understand the institutional modalities for managing and delivering healthcare services in the Northern Macroregion of Paraná, Brazil. Among the institutional modalities identified in the region, this subset focuses on “philanthropic entities.”

Initially, an exploratory research was conducted using secondary data available in the National Registry of Health Facilities (SCNES) database. The analysis focused on the evolution of the number of “non-profit” entities from 2012 to 2020 in the studied region, at both the state and national levels.

After identifying philanthropic institutions in the region, the study selected services that were presented as a local or regional strategy for specialized care, which constituted the case study.

A semi-structured interview script was used to gather data from key informants, including municipal health administrators, directors of philanthropic healthcare service entities, regional health technicians, state management representatives, members of the Council of Municipal Health Departments (Cosems), and members of social control related to those institutions. A total of 22 interviews were conducted from December 2019 to January 2021.

The secondary data provided a general understanding of the phenomenon and its representation in the studied region, the state of Paraná, and the country. The empirical material derived from the case study was subjected to critical hermeneutic analysis, interpreted with the support of policy analysis framework^6 , 7^ . The research was approved by the Ethics Committee to which researchers are affiliated, under opinion no. 4.074.080.

## RESULTS AND DISCUSSION

From 2012 to 2020, there was an increase of 30% of non-profit entities in Brazil^8^ . This trend was identified in all Brazilian geographical regions ( Figure 1), with greater emphasis on the Southeast region, which may be related to its higher concentration of private services^9^ .

**Figure:**
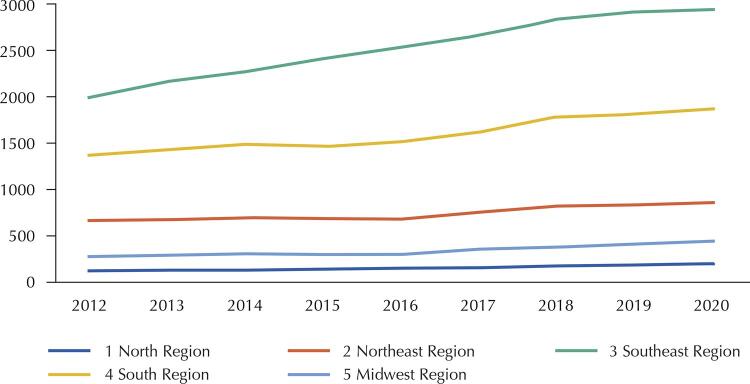
Evolution of the number of non-profit entities (private associationa) in Brazilian regions, 2012–2020.

^a^ Name of non-profit health care entities in the SCNES.

Source: SCNES ^8^ .

In the macro-region studied, philanthropic entities correspond to 36% of entities and allocate around 65% of SUS beds^8^ . Over the analyzed period, there was a decline in the number of “for-profit” private entities, whereas “non-profit” entities increased by 20% in the state where the study region is located. This trend aligns with the local government’s gradual implementation of policies specifically targeted at supporting non-profit entities.

The institutions investigated through the case study were classified into two groups: pure philanthropic and private philanthropic. Pure philanthropic institutions include entities designed with a philanthropic mission, whereas private philanthropic institutions are private entities that have changed their legal nature for strategic purposes.

The case study confirmed a process of changing the legal nature of private healthcare entities from “for-profit” to “non-profit” status. The analysis of empirical material revealed that this process may be related to several factors such as: induction promoted by tax and fiscal exemptions offered by this change; the possibility of entering into agreements, having priority regarding the complementarity of services and/or being eligible to request and receive parliamentary amendments and contractual incentives from public management; and strategic analysis of the viability of these entities in a context of financial constraints.

Another important finding from the field research was the differences between the scope of action of “pure” and “private” philanthropic entities. The former, depending on their size, have a certain regional insertion and are entities willing to offer services to the Unified Health System (SUS). On the other hand, “private” philanthropic entities behave like for-profit organizations, but benefit from legal exemptions, practice supply-side economics, allow differentiated access to users from certain contracted municipalities, are not open to the public, and occasionally manage supplementary services. In some cases, the previous owners rent the property to the entity and are presidents of the associations, so this category can also be identified as “philanthropic with owners.” The participants’ statements express these results:

“Nothing has changed [...] it’s still the same, there is only ‘Associação Mais Saúde,’ the name changed, that’s all” (E9).

“No benefit [for the municipality], it is the same. For us, the only thing that changed was the CNPJ” (E14).

“When we became a Foundation [philanthropic], the population did not notice it, there are a lot of people who do not know or understand it, but again, it would not change anything [...]” (E8).

These aspects may represent an expansion of the commodification of public health services through non-profit private institutions^1^ . This is an implicit commodification process characterized by the growing adoption of private sector practices by the public sector^5^ . In Brazil, this is already a reality observed in different regions of the country, so the phenomenon of changing the legal nature from “for-profit” to “non-profit” entities is confirmed through a series of policies aimed at this group, with specific contracting programs and support through financial incentives.

The case study also identified other important elements of this scenario that deserve to be highlighted, including:

- public-private relationships established through the involvement of market actors (providers) and different public actors, including legislators, in defining contractual terms;
- private providers with greater decision-making power regarding the provision of services to the public system;
- limited mechanisms for regulating services by public management;
- political-party relationships between board members of these associations and local public actors; and
- strengthening of health service outsourcing.

It should be noted that, depending on the characteristics of the municipal budget management for the federal ceiling of medium and high complexity, the negotiation of parliamentary amendments can occur directly through the institution, without any guarantee that they will be used for the benefit of public system users. Furthermore, they may gather resources based on their legal nature which, due to a lack of proper transparency, may not always be used for social benefits.

## FINAL CONSIDERATIONS

The change in legal nature of private healthcare entities from “for-profit” to “non-profit” status in Brazil hides a broader process of commodification of health services and represents a strategic action by market actors. Empirical data also suggest that the increase of philanthropic entities in the Brazilian Unified Health System (SUS) does not reflect in the quality of healthcare provision.

These institutions maintain the behavior of a for-profit private institution, but with the benefits of philanthropic institutions. Due to a lack of state regulation, these institutions maintain low levels of transparency regarding the percentage of services provided to the public, as well as distinct physical and structural organizations for public and private users.

In this sense, the importance of research within this theme is perceived in order to verify its expression in different regions. Furthermore, policies are recommended to minimize disparities in the delivery of services by philanthropic entities through criteria that enhance popular participation, regional contractual specificities, comprehensive care, and mechanisms for regulating and governing public entities. These policies should prioritize services that meet the needs of the population, rather than favoring interests of the market.
