# Supplementary material for: The increase of philanthropic entities in the SUS: what does this scenario reveal?
Source: Rev Saude Publica. 2023 Jun 2;57:34. doi: 10.11606/s1518-8787.2023057004720 (PMC10317140; doi:10.11606/s1518-8787.2023057004720)
Supplement: Translation

# Translation: O aumento de entidades filantrópicas no SUS: o que esse cenário revela?

**Authors:** João Felipe Marques da Silva, Brígida Gimenez Carvalho, Adelyne Maria Mendes Pereira, Elisabete de Fátima Almeida Nunes, Fernanda de Freitas Mendonça, Stela Maris Lopes Santini, Silvia Karla Azevedo Vieira Andrade, Edinalva de Moura Ferraz

**Affiliations:** I Brasil Universidade Estadual de Londrina. Programa de Pós Graduação em Saúde Coletiva. Londrina, PR, Brasil; II Brasil Universidade Estadual de Londrina. Centro de Ciências da Saúde. Departamento de Saúde Coletiva. Londrina, PR, Brasil; III Brasil Fundação Oswaldo Cruz. Escola Nacional de Saúde Pública Sergio Arouca. Departamento de Administração e Planejamento em Saúde. Rio de Janeiro, RJ, Brasil; IV Brasil Fundação Oswaldo Cruz. Escola Nacional de Saúde Pública Sergio Arouca. Programa Pós-Doutorado Junior - Inova Fiocruz. Rio de Janeiro, RJ, Brasil

**Keywords:** Instituições Filantrópicas de Saúde, legislação & jurisprudência, Obtenção de Fundos, Gestão em Saúde, Sistema Único de Saúde, Política Pública

## Abstract

O artigo analisa aspectos da mudança da natureza jurídica de instituições privadas de assistência à saúde, de estabelecimentos “com” para “sem” fins lucrativos. Trata-se de uma pesquisa exploratória, apoiada no referencial de análise de políticas, com foco em dados secundários, provenientes do Sistema de Cadastro Nacional de Estabelecimentos de Saúde (SCNES), de 2012 a 2020, e estudo de caso. Os resultados apresentam aumento dessas entidades em todas as regiões do país e evidências de que se comportam como estabelecimentos com fins lucrativos. A mudança de natureza jurídica oculta um processo mais amplo de mercantilização implícita dos serviços de saúde, incentivado por políticas estatais e relacionado às isenções previstas em lei.

## INTRODUÇÃO

No Brasil, as instituições privadas sem fins lucrativos são responsáveis por expressiva parcela da atenção especializada no país^1^ e compõem um campo da complexa relação público-privada no Sistema Único de Saúde (SUS). Esta relação é marcada por assimetrias de poder quanto ao processo decisório e de governança da política de saúde^1 , 2^ , e inserida em um histórico de fomento, por parte do Estado, à expansão do setor privado. Tal prática pode se configurar pela mercantilização dos serviços de saúde, valendo-se de subsídios diretos e indiretos (isenções fiscais e tributárias) aplicados a essas entidades^3^ .

A mercantilização se relaciona ao aumento da lógica privada, mediante a participação direta do setor privado, enquanto prestador de serviços de saúde, e a adoção de princípios de gestão, remuneração e organização dos sistemas^4^ . Tais características parecem estimular a mudança da natureza jurídica de estabelecimentos privados “com” para “sem” fins lucrativos.

Esse processo tem propiciado novas relações público-privadas, de caráter neopatrimonialista, devido aos benefícios que envolvem, na atualidade, o Estado e o mercado. Além disso, as políticas de contrarreforma da década de 1990 incentivaram o fortalecimento de modalidades que se beneficiam com as prerrogativas legais das entidades sem fins lucrativos^5^ .

Este estudo tem como foco a mudança da natureza jurídica das instituições prestadoras de serviços de saúde nos últimos dez anos. Baseia-se em dois argumentos principais: o primeiro refere-se a uma transição de estabelecimentos privados “com” para “sem” fins lucrativos, em um processo que pode representar uma tendência de mudança nas relações público-privadas na saúde no Brasil; e o segundo, vinculado ao anterior, diz respeito a mecanismos de mercantilização que podem estar sendo empregados por essa via. Nessa perspectiva, visa analisar e identificar características desse fenômeno, com o intuito de fomentar e ampliar o debate, bem como trazer proposições e estimular outras análises a respeito do tema.

## MÉTODOS

Trata-se de um recorte de uma tese de doutorado, que buscou compreender as modalidades institucionais de gestão e prestação de serviços na Macrorregião Norte do Paraná. Dentre as modalidades institucionais identificadas na região, este recorte teve como foco a “entidade filantrópica”.

Inicialmente, realizou-se pesquisa exploratória, a partir de dados secundários disponíveis na base do Sistema de Cadastro Nacional de Estabelecimentos de Saúde (SCNES), por meio da análise da evolução do número de entidades “sem” fins lucrativos entre 2012 e 2020, na região estudada, nos níveis estadual e nacional.

Por meio da identificação das instituições filantrópicas desta região, selecionaram-se os serviços que se apresentavam como estratégia local ou regional para a assistência especializada, constituindo-se o caso em estudo.

Foram entrevistados, por meio de roteiro semiestruturado, a partir da técnica de informantes-chave, os gestores municipais de saúde, diretores das instituições filantrópicas prestadoras de serviços, técnicos regionais de saúde, representantes da gestão estadual, representantes do Conselho de Secretarias Municipais de Saúde (Cosems) e membros do controle social que se relacionavam àquelas instituições, totalizando 22 entrevistas entre dezembro de 2019 e janeiro de 2021.

Os dados secundários permitiram uma compreensão geral do fenômeno e sua representação na região estudada, no estado do Paraná e no país. O material empírico derivado do estudo de caso foi submetido à análise hermenêutica crítica, sendo interpretado com apoio do referencial de análise de políticas^6 , 7^ . A pesquisa foi aprovada pelo Comitê de Ética ao qual os pesquisadores estão vinculados sob o parecer nº 4.074.080.

## RESULTADOS E DISCUSSÃO

Entre os anos de 2012 e 2020, observou-se um incremento de 30% de estabelecimentos sem fins lucrativos no Brasil^8^ . Essa tendência foi identificada em todas as regiões geográficas brasileiras ( Figura 1), com maior destaque para a região Sudeste, o que pode estar relacionado ao fato de esta apresentar uma maior concentração de serviços privados^9^ .

**Figura:** Evolução do número de entidades sem fins lucrativos (associação privadaa) em regiões brasileiras, 2012–2020.

^a^Denominação de entidades de assistência à saúde sem fins lucrativos no SCNES.

Fonte: SCNES ^8^ .

Na macrorregião estudada, as instituições filantrópicas correspondem a 36% dos estabelecimentos e alocam cerca de 65% dos leitos do SUS^8^ . No período sob análise, constatou-se uma redução das entidades privadas “com” fins lucrativos e aumento em 20% do número de estabelecimentos de natureza jurídica “sem” fins lucrativos no estado em que a região estudada está inserida, fato que coincide com a publicação gradativa de políticas específicas pelo governo local para tais estabelecimentos.

As instituições investigadas por meio do estudo de caso foram classificadas em dois grupos: filantrópicos puros e filantrópicos privados. Os estabelecimentos identificados como filantrópicos puros, ou originalmente filantrópicos, incluem instituições concebidas com a missão da filantropia; e os estabelecimentos classificados como filantrópicos privados são estabelecimentos privados que alteraram sua natureza jurídica com fins estratégicos.

O estudo de caso confirmou um processo de mudança da natureza jurídica das instituições privadas “com” para “sem” fins lucrativos. A análise do material empírico evidenciou que esse processo pode estar relacionado a alguns fatores como: indução promovida pelas isenções fiscais e tributárias que essa alteração oferece; possibilidade de firmar convênios, ter preferência quanto à complementaridade de serviços e/ou estar apto para solicitar e receber emendas parlamentares e incentivos contratuais da gestão pública; e análise estratégica acerca da viabilidade desses estabelecimentos em um contexto de restrições financeiras.

Destaca-se outro importante achado da pesquisa de campo: diferenças entre o escopo de atuação dos estabelecimentos filantrópicos “puros” e “privados”. Os primeiros, a depender de seu porte, possuem determinada inserção regional e são estabelecimentos dispostos à oferta de serviços ao SUS. Já os filantrópicos “privados” comportam-se como estabelecimentos com fins lucrativos, mas se beneficiam de isenções legais, praticam gerencialismo da oferta, possibilitam acesso diferenciado aos usuários de determinados municípios contratados, não são porta aberta à população e eventualmente fazem a gestão de serviços suplementares. Em alguns casos, os antigos donos alugam o imóvel para a própria entidade e são os presidentes das associações, de modo que essa categoria também pode ser identificada como “filantrópicos com donos”. As falas dos participantes expressam esses resultados:

“Não [mudou] nada […] continua do mesmo jeito, só tem lá ‘Associação Mais Saúde’, o que mudou foi o nome mesmo” (E9).

“Nenhum benefício [para o município], do mesmo jeito. Para nós o que mudou foi o CNPJ” (E14).

“Quando a gente passou para Fundação [filantrópica], a população não percebeu isso, tem muita gente que não sabe, não conhece isso, mas também, não ia mudar nada […]” (E8).

Tais aspectos podem representar uma ampliação da mercantilização dos serviços públicos de saúde por meio de instituições privadas sem fins lucrativos^1^ . Trata-se de um processo de mercantilização implícita, caracterizado pela adoção crescente da lógica de atuação privada por parte do setor público^5^ . No Brasil, essa já é uma realidade observada nas diferentes regiões do país, de modo que o fenômeno da alteração da natureza jurídica de “com” para “sem” fins lucrativos se confirma através de uma série de políticas destinadas a esse grupo, com programas específicos de contratualização e apoio por meio de incentivos financeiros.

O estudo de caso também identificou outros elementos importantes desse cenário que merecem ser destacados, entre eles:

- relação público-privada constituída por meio do envolvimento entre atores de mercado (prestadores) e diferentes atores públicos, incluindo parlamentares, na definição dos aspectos contratuais;
- prestadores privados com maior poder de decisão quanto à oferta de serviços ao sistema público;
- incipientes mecanismos de regulação dos serviços pela gestão pública;
- relação político-partidária entre membros da diretoria dessas associações e atores públicos do nível local; e
- fortalecimento da terceirização de serviços de saúde.

Cabe destacar que, a depender das características da gestão orçamentária municipal do teto federal de média e alta complexidade, a negociação de emendas parlamentares pode ocorrer diretamente pela instituição, sem a garantia de que serão utilizadas em favor dos usuários do sistema público. Ademais, podem angariar recursos em função de sua natureza jurídica que, por ausência de devida transparência, nem sempre serão utilizados em benefícios sociais.

## CONSIDERAÇÕES FINAIS

A mudança da natureza jurídica de instituições privadas “com” para “sem” fins lucrativos no Brasil oculta um processo mais amplo de mercantilização dos serviços de saúde e representa uma ação estratégica dos atores de mercado. Os dados empíricos também permitem inferir que o aumento das entidades filantrópicas no SUS parece não repercutir na qualidade da assistência à saúde.

Estas instituições mantêm o comportamento de uma instituição privada lucrativa, mas com os benefícios de instituições filantrópicas. Esse fenômeno é favorecido pela incipiente regulação estatal, e as instituições se mantêm com baixa transparência quanto ao percentual de serviços prestados ao público, e distintas organizações físicas e estruturais do serviço para recepção dos usuários públicos e privados.

Nesse sentido, percebe-se a importância de pesquisas dentro dessa temática, a fim de verificar sua expressão em diferentes regiões. Além disso, advoga-se por políticas que minimizem as iniquidades quanto à oferta de serviços das entidades filantrópicas por meio de critérios que fortaleçam a participação popular, as especificidades contratuais regionais, a integralidade da atenção e os mecanismos de regulação e governança dos entes públicos, na perspectiva de serviços voltados às necessidades da população, e não aos interesses do mercado.
